# Clinical characteristics according to the laterality of ocular torsion in unilateral superior oblique palsy

**DOI:** 10.1186/s12886-018-0977-x

**Published:** 2018-12-17

**Authors:** Ju-Yeun Lee, Hyo Jeong Kim, Kyung-Ah Park, Shin Yeop Oh, Sei Yeul Oh

**Affiliations:** 1 Department of Ophthalmology, Samsung Medical Centre, Sungkyunkwan University School of Medicine, #81 Irwon-ro, Gangnam-gu, Seoul, 06351 South Korea; 2 Department of Ophthalmology, Samsung Chanwon hospital, Sungkyunkwan University School of Medicine, Chanwon, South Korea

**Keywords:** Horizontal strabismus, Superior oblique palsy, Ocular torsion, Discordance, Excyclotorsion

## Abstract

**Background:**

To compare clinical characteristics according to the laterality of objective ocular torsion in patients with unilateral superior oblique palsy (SOP).

**Methods:**

This retrospective study included all patients with a diagnosis of unilateral SOP. They were classified into subgroups according to correspondence between the paretic eye and the extorted eye using fundus photography. Ocular alignment and muscle action were tested by the prism and alternate cover tests and 4-scale movement measure. Various clinical factors, including the amount of preoperative ocular torsion and change in ocular torsion postoperative, were compared between the accordance and disaccordance groups.

**Results:**

A total of 70 Asian patients (140 eyes) were included and underwent fundus photography preoperatively. Excyclotorsion in the paretic eye was defined as accordance (45 patients), excyclotorsion in the nonparetic eye was defined as disaccordance (25 patients). The presence of horizontal strabismus was detected in 28 (62%) patients in the accordance group and only 8 (32%) patients in the disaccordance group (*p* = 0.024). All horizontal strabismus observed in the accordance group involved exodeviation. The proportion of horizontal strabismus surgery was also significantly larger in the accordance group than the disaccordance group (*p* = 0.039). Among those patients, there were 26 who underwent fundus photography postoperatively. There was significant reduction in ocular excyclotorsion postoperatively in the accordance group (*p* = 0.001), but no significant reduction postoperatively in the disaccordance group (*p* = 0.270). There was no significant correlation between the amount of torsional reduction and the amount of vertical deviation reduction (*p* = 0.979).

**Conclusions:**

In cases of preoperative excyclotorsion in paretic eyes, careful consideration of combined horizontal misalignment which may require surgical correction is helpful to manage unilateral SOP.

## Background

Superior oblique muscle palsy (SOP) is common cranial nerve palsy that may be caused by trauma, vascular disease, or congenital conditions [1]. Patients with a unilateral SOP typically have hypertropia of the paretic eye that increases on ipsilateral head tilt [2]. In addition, excyclotorsion in the paretic eye is one of the features in SOP.

Many authors have investigated the significant ocular excyclotorsion in paretic eyes and reduction of excyclotorsion after surgery for SOP [3, 4]. Although patients with a unilateral SOP typically have an excyclotorsion in the paretic eye, excyclotorsion of the non-paretic fellow eye has been reported [5, 6]. Na et al. reported 25% of ocular excyclotorsion in non-paretic eyes in unilateral SOP, and Lee et al. reported 38.7% of this discordance in acquired SOP. Because this seemingly paradoxical test result can complicate the diagnosis of SOP, we believed that it deserved further clinical study. In addition, there is few study to focus the clinical implication of this paradoxical result. Therefore, we aimed to evaluate clinical implication of objective ocular torsion and to compare various clinical factors between the subgroups according to the laterality of objective ocular torsion in unilateral SOP.

## Methods

### Patients

This hospital-based retrospective observational study was a single-centre study from 2010 to 2015 that was conducted in accordance with the tenets of the Declaration of Helsinki. The study was approved by the ethics committee of the Samsung Medical Centre Institutional Review Board. Patient records were anonymised and de-identified prior to analysis.

All subjects with a diagnosis of unilateral SOP who underwent primary inferior oblique weakening surgery were included. SOP was diagnosed based on a history of ocular torticollis and/or vertical misalignment and fulfilment of the Park’s 3-step test. Diagnosis of the congenital superior oblique palsy was determined by photographic or historical documentation of a head tilt since early childhood, facial asymmetry, and increased vertical fusional amplitudes. Patients with acquired superior oblique palsy were identified by the lack of the aforementioned findings and a history of a previous event related to the onset of symptoms.

Patients who had eyes with anatomical abnormalities and those with a history of previous ocular surgery, other paralytic strabismus except SOP, congenital cataract, other retinal disorders, craniosynostosis, any other neurologic deficits, or developmental delay were excluded from this study. The goal of the surgery was to correct the hypertropia and head tilt; patients underwent surgery based on the amount of hypertropia or head tilt. Masked bilateral SOP patients in whom development of inferior oblique over action (IOOA) in the unoperated eye following unilateral IO muscle weakening surgery were also excluded.

### Measurements

All patients underwent complete ophthalmic examinations, including the prism and alternate cover test, and Bielschowsky’s head tilt test. Ocular alignment was tested by the prism and alternate cover tests, which were carried out at distances of 6 m in nine cardinal gazes and 30 cm in primary gaze. The amount of deviation in primary gaze and the maximal amount of deviation were used for analysis. Oblique muscle dysfunction was subjectively graded on a scale from − 4 (maximum underaction) to + 4 (maximum overaction). Each patients was examined at least three times (at least during 6 months) before surgery to ascertain stability of ocular deviation. In patients who had significant vertical deviation with IOOA, head tilt to non-paretic side, facial asymmetry, or vertical diplopia, paretic eye was considered to undergo surgery.

We measured objective ocular torsion of both eyes using fundus photography obtained by a fundus camera (Topcon Medical System, Tokyo, Japan) just before the surgery [4, 7]. We additionally used a cervical range of motion (CROM) device (Performance Attainment Associates, Roseville, MN, USA) for accurate control of head tilt. While obtaining fundus photographs, the examiner held each subject’s head and watched the CROM angle to make sure subjects were maintaining the correct angle. The detailed methodology for these measurements has been described in our previous studies [8]. The postoperative fundus photographs were obtained at least 3 months after the surgery. The patients asked to look at an internal fixation target to align their eyes in the primary position, and each eye was performed separately with being fixed. Poorly cooperative subjects were excluded from data analysis.

**Fig. 1:**
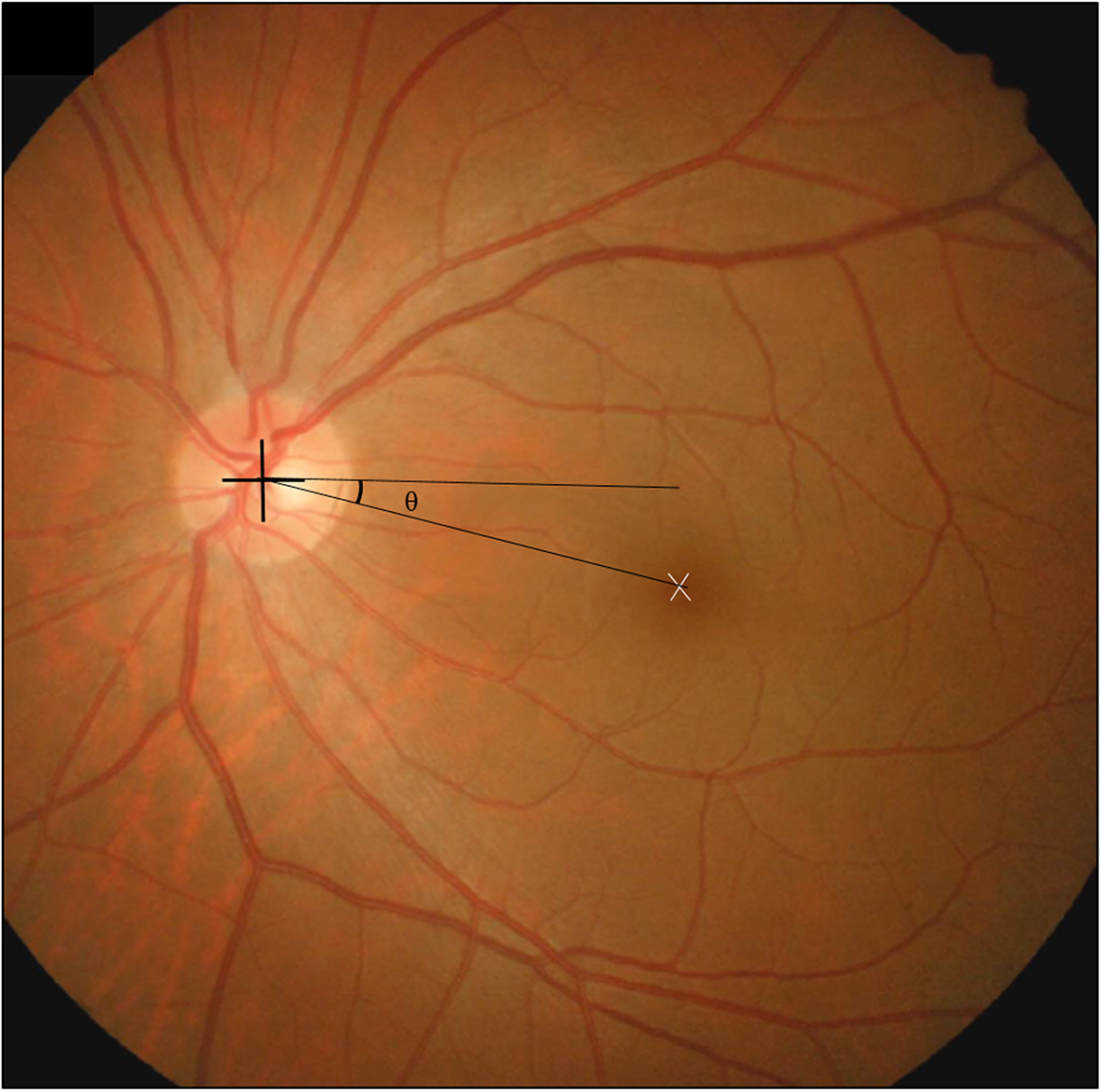
Disc-foveal method to measure ocular torsion in patients with unilateral superior oblique palsy

The disc-foveal angle was calculated from a single well-focused photograph using the National Institutes of Health image analysis software (ImageJ 1.42q; developed by Wayne Rasbands, National Institutes of Health, Bethesda, MD, USA) (Fig. 1) [7, 9]. Based on the method, the amount of ocular torsion were recorded. Two experienced examiners (J.L, H.K) who were blinded as to whether the eye is paretic or not, analysed each image. They measured twice for all data, and the mean value of measurements was used for analysis.

All patients were classified into subgroups according to correspondence between the paretic eye and the more extorted eye, as evaluated on fundus photography. All patients were divided into accordance (excyclotorsion in the paretic eye) and disaccordance (excyclotorsion in the contralateral eye) groups. Those without torsion in either eye or excyclotorsion in both eyes were excluded from analysis. Data on sex, age, age at surgery, laterality of SOP, type of SOP (congenital/acquired), duration of strabismus, best corrective visual acuity (BCVA), refractory error, fixation preference eye, preoperative angle of deviation (prism diopter; PD), presence and grade of IOOA, grade of SOP, and presence of horizontal strabismus, amblyopia, or disassociated vertical deviation (DVD) were collected. Various clinical factors including the amount of preoperative and postoperative ocular torsion and change in ocular torsion were compared between the accordance and disaccordance groups. The correlation between amount of ocular torsion and each clinical parameter was also analysed.

### Primary outcome measure

Preoperative ocular torsion in the accordance and disaccordance groups, preoperative angle of deviation and presence of horizontal strabismus are primarily collected for the primary analysis.

### Statistical analyses

Statistical analyses were performed by an independent statistician. Data were analysed using the Statistical Analysis System (SAS Institute, Cary, NC, USA). The intra- and inter-observer reproducibility values were analyzed using an intraclass correlation coefficient. Kruskal-Wallis test and Fisher’s exact test were used to compare clinical parameters and Mann-Whitney U test and Fisher’s exact test were used to sub-analyse clinical parameters between the accordance and disaccordance groups. Spearman correlation and Wilcoxon rank sum test were used to analyse the correlations between ocular torsion and clinical factors. Statistical differences were considered significant when the *p* value was less than 0.05. Results are expressed as means ± standard deviations.

## Results

We enrolled 157 patients who met the eligibility criteria. Four patients did not complete the follow up visit, 37 patients did not undergo fundus photography (including those that were uncooperative with fundus photography due to younger age), 31 patents had no torsion in either eye, 15 patents had excyclotorsion in both eyes in fundus photography. Therefore, a total of 70 patients (140 eyes), including 32 males and 38 females, were finally included. Forty-five patients were enrolled in the accordance group and 25 patients in the disaccordance group. Among a total of 56 patients with congenital SOP, 36 (64%) were in the accordance group and 20 (36%) in the disaccordance group. Among 14 patients with acquired SOP, 9 (64%) were in the accordance group, 5 (36%) in the disaccordance group. Patient demographics are presented in Table 1.

**Table 1:** Patient demographics and clinical factors

*Variable*	Congenital (*n* = 56)	Acquired (*n* = 14)	*p-*value
	Mean ± SD	Mean ± SD	
Age (years)	11 ± 6	41 ± 12	< 0.001^a^
Gender (Male/Female)	38/18	11/3	0.524^b^
Age at surgery (years)	8 ± 6	34 ± 12	< 0.001^b^
Laterality (Right/Left)	25/31	5/9	0.764 ^a^
BCVA of paretic eyes (logMAR)	0.92 ± 0.11	0.89 ± 0.14	1.000 ^a^
Refractive error of paretic eyes (PD)	−0.78 ± 2.20	−1.05 ± 1.71	0.236 ^a^
Grade of inferior oblique overaction	2.1 ± 0.6	0.9 ± 0.6	< 0.001^a^
Grade of superior oblique palsy	1.2 ± 0.7	1.1 ± 0.5	0.659 ^a^
Presence of horizontal strabismus	32 (57%)	3 (21%)	0.017^b^
Presence of disassociated vertical deviation	3 (5%)	0	1.000^b^
Preoperative vertical deviation at primary gaze (PD)	8.8 ± 6.1	8.7 ± 8.1	0.614^a^
Preoperative maximal vertical deviation (PD)	15.4 ± 7.9	15.5 ± 8.4	0.440^a^
Laterality of cyclodeviation			
Excyclotorsion in the paretic eyes	36 (64%)	9 (64%)	1.000^b^
Excyclotorsion in the nonparetic eyes	20 (36%)	5 (36%)	1.000^b^

^a^Wilcoxon Mann-Whitney test

^b^Fisher’s exact test

*BCVA* best-corrected visual acuity;

### Comparison of clinical factors between ‘excyclotorsion in the paretic eye’ and ‘excyclotorsion in the contralateral eye’

Among clinical factors, the presence of horizontal strabismus showed a significant difference between the accordance and disaccordance groups (*p* = 0.024) (Table 2). Most of parameters comprising age, age at surgery, laterality of SOP, type of SOP, duration of strabismus, BCVA, refractory error, fixation preference eye, preoperative angle of deviation, grade of IOOA and SOP, stereoacuity, amblyopia, DVD and the presence of head tilt showed no significant difference between the accordance and disaccordance groups (*p* > 0.05, all parameters).

**Table 2:** Clinical factors between the accordance (excyclotorsion in the paretic eye) and disaccordance (excyclotorsion in the nonparetic eye) groups

*Variable*	Accordance (*n* = 45)	Disaccordance (*n* = 25)	*p*-value
	Mean ± SD	Mean ± SD	
Age (years)	17 ± 14	17 ± 16	0.786^a^
Gender (Male/Female)	32/13	16/9	0.597^b^
Age at surgery (years)	9 ± 8	12 ± 11	0.239^a^
Laterality (Right/Left)	15/30	15/10	0.044^b^
Type (Congenital/Acquired)	36/9	20/5	1.000^b^
BCVA (logMAR)	0.90 ± 0.17	0.92 ± 0.12	0.836^a^
Refractive error (Prism diopter)	−0.92 ± 2.14	−0.68 ± 2.10	0.781^a^
Grade of inferior oblique overaction	1.8 ± 0.8	2.0 ± 0.8	0.993^a^
Grade of superior oblique palsy	1.2 ± 0.6	1.3 ± 0.7	0.212^a^
Horizontal strabismus	28 (62%)	8 (32%)	0.024^b^
Disassociated vertical deviation	2 (4%)	1 (4%)	1.000^b^
Preoperative angle of deviation (PD)	7.6 ± 5.1	10.0 ± 8.8	0.170^a^
Postoperative angle of deviation (PD)	0.4 ± 0.8	0.7 ± 1.1	0.660^a^
Good preoperative stereoacuity (better than 100 arc/sec)	10 (22%)	8 (32%)	1.000^†^

^a^Wilcoxon Mann-Whitney test

^b^Fisher’s exact test

*BCVA* best-corrected visual acuity;

Based on the primary results, we sub-analyzed clinical factors between the accordance and disaccordance groups. The presence of horizontal strabismus was detected in 28 (62%) patients in the accordance group and 8 (32%) patients in the disaccordance group. All patients in the accordance group had combined exodeviation. Of these, 21 (47%) patients underwent strabismus surgery for horizontal deviation. In the disaccordance group, 6 (75%) patients had exodeviation and 2 (25%) patients had esodeviation. Of these, 5 (20%) patients underwent strabismus surgery for horizontal deviation. The proportion of horizontal strabismus surgery showed a significant difference (*p* = 0.039).

The proportion of ocular excyclotorsion that was consistent with fixation preference was 56% in the accordance group and 50% in the disaccordance group with no significant differences (Chi-square test, *p* = 0.513). Pre and postoperative angle of vertical deviation was not significantly different between the accordance and disaccordance groups. (*p* = 0.170, and 0.660 respectively).

### Postoperative change in ocular excyclotorsion

Among patients in the accordance and disaccordance groups, there were 26 who underwent fundus photography postoperatively, 14 patients in the accordance group and 12 patients in the disaccordance group. In the accordance group, preoperative excyclotorsion was 13.8 ± 6.8 degrees and postoperative excyclotorsion was 6.8 ± 4.6 degrees. There was significant reduction in ocular excyclotorsion measured (paired-t, *p* = 0.001). In the disaccordance group, preoperative excyclotorsion was 12.2 ± 4.2 degrees, and postoperative excyclotorsion was 10.8 ± 5.0 degrees. There was no significant reduction in ocular excyclotorsion after surgery (paired-t test, *p* = 0.270). The mean amount of postoperative reduction in ocular excyclotorsion was 6.5 ± 4.6 degrees in the accordance group, and 1.3 ± 3.7 degrees in the disaccordance group. There was a significant difference in the amount of reduction between the accordance and disaccordance groups (Mann-Whitney test, *p* = 0.001). There was no significant correlation between the amount of torsional reduction and the amount of primary/maximal vertical deviation reduction (Spearman correlation, *p =* 0.979 and 0.487, respectively).

### Correlations between ocular torsion and clinical factors

The correlations between the amount of excyclotorsion and various clinical factors were analyzed (Table 3). The amount of ocular excyclotorsion measured was significantly correlated with grade of IOOA and maximal amount of hypertropia (on head tilt) (*p* = 0.024, and 0.010, respectively). Whereas, ocular excyclotorsion showed no significant correlation with grade of SOP or amount of hypertropia at primary gaze (*p* > 0.05, all parameters).

**Table 3:** Correlation analysis between the amount of preoperative ocular torsion measured by fundus photography and clinical factors

*Variable*	*Ocular torsion (Disc-foveal angle)*	
	Correlation coefficient	*p-*value
Age at surgery	−0.007	0.945^b^
Gender		0.749^a^
Laterality of SOP		0.483^a^
Type of SOP		0.777^a^
Visual acuity in paretic eyes	−0.148	0.142^b^
Visual acuity in nonparetic eyes	−0.068	0.500^b^
Spherical equivalent	0.184	0.062^b^
Primary vertical deviation	0.106	0.278^b^
Maximal vertical deviation	0.244	0.012^b^
IO overaction grade	0.394	0.047^b^
SO underaction grade	−0.032	0.746^b^
Stereopsis (Good/Bad)		0.778^a^
Fixation preference		0.317^a^
The presence of amblyopia		0.383^a^
The presence of horizontal strabismus		0.852^a^
The presence of DVD		0.223^a^
The presence of head tilt		0.947^a^
Type of IO weakening surgery		0.411^a^

^a^ Wilcoxon rank sum test

^b^ Spearman correlation analysis

*SOP* superior oblique palsy, *IO* inferior oblique, *SO* superior oblique, *DVD* disassociated vertical deviation

The intra-examiner reproducibility value was 0.97 (95% confidence interval [CI]: 0.96–0.99), and the inter-examiner reproducibility value was 0.87 (95% CI: 0.85–0.92). The post-hoc power calculation showed that this study had an 80% or greater power to evaluate the difference in ocular torsion given the standard deviation of torsional measurements among the subjects.

## Discussion

Excyclotorsion in SOP is caused by paralysis of the anterior fibers of the SO muscle, thus significant ocular cyclodeviation is usually observed in paretic eyes. In contrast to this expectation, some studies have demonstrated paradoxical ocular excyclotorsion in non-paretic eyes of unilateral SOP. Lee et al. reported cyclotorsion of non-paretic eyes in 12 patients; they also found that objective torsion was greater than subjective torsion [5]. We demonstrated a 21% discrepancy between paretic eyes and ocular cyclodeviation, in accordance with previous studies [6, 10].

Based on the primary outcomes, we determined that horizontal strabismus was more frequently observed and corrected in patients who show significant excyclotorsion in their paretic eyes, and all patients in the accordance group showed exodeviation. Our results were similar to a previous study by Telander et al., reporting 59% of horizontal misalignment in patients with unilateral SOP [11]. This result can be explained by the secondary overaction of the IO muscle which can directly affect the development of exodeviation in paretic eyes, but may not directly affect non-paretic eyes. We also observed that the proportion of exodeviation were larger in congenital SOP than in acquired SOP. This might be due to stronger action of the IO muscle in congenital SOP compared with in acquired SOP, as there were significantly higher grade of IOOA in congenital type. Therefore, careful consideration of combined exodeviation which can require surgical correction in patients who show significant excyclotorsion in their paretic eyes, is helpful to establish therapeutic plans for unilateral SOP. 1.

It is interesting to note that postoperative changes of ocular torsion were larger in the accordance group than the disaccordance group, consistent with the results by Na et al. [6]. In the accordance group, a significant reduction in ocular torsion of paretic eyes may be caused by postoperative mechanical changes in extraocular muscles which has a direct effect on torsional force. On the other hand, a non-significant reduction in non-paretic eyes could be a result of the secondary repositioning of the retinal elements according to change in adaptation of the paretic eye postoperatively in the disaccordance group. This secondary adaptation, which has a minor effect on torsional reduction, may be insufficient to reduce excyclotorsion significantly. In contrast to the different amounts of reduction in ocular torsion, the postoperative angle of deviation was comparable. Hence, the results indicate that the presence of significant torsional reduction may not be related with postoperative outcomes in unilateral SOP.

There were several limitations to this study. First, this was a retrospective study and all patients were of the same ethnicity. Second, we measured ocular torsion with fundus photography, which could have possible sources of interpretation error according to fixation or gaze direction. We used CROM device controlling subjects’ head position, and cautioned against improper head positioning in order to minimize the effect of error from gaze direction. In addition, based on the results of study by Kim et al. reporting no significant difference in ocular torsion according to fixation, we could rule out possible error according to fixation in interpreting the results. Second, the number of patients undergoing fundus photography postoperatively is small, and it could undermine the postoperative results. Therefore, further well controlled studies with larger sample size are needed for verifying our results. Finally, we did not evaluate subjective ocular torsion and could not suggest sensorial outcomes related to the paradoxical torsional results.

In conclusion, we demonstrated a possible relationship between the laterality of ocular torsion in unilateral SOP and other clinical factors. Based on these results, in cases of unilateral SOP in those with accordance or congenital SOP, careful evaluation of combined exodeviation is recommended.

## Abbreviation

BCVA: Best corrected visual acuity
CROM: Cervical range of motion
DVD: Disassociated vertical deviation
IOOA: Inferior oblique over action
PD: Prism diopter
SOP: Superior oblique palsy
